# 2019 Influenza Vaccination Campaign in an Italian Research and Teaching Hospital: Analysis of the Reasons for Its Failure

**DOI:** 10.3390/ijerph17113881

**Published:** 2020-05-30

**Authors:** Manuel Maffeo, Ester Luconi, Ambra Castrofino, Emanuela Maria Campagnoli, Andrea Cinnirella, Federica Fornaro, Claudia Gallana, Pier Mario Perrone, Viktoriia Shishmintseva, Elena Pariani, Silvana Castaldi

**Affiliations:** 1 Postgraduate School in Public Health, Department Biomedical Sciences for Health, University of Milano, 20136 Milano, Italy; ambra.castrofino@unimi.it (A.C.); emanuela.campagnoli@unimi.it (E.M.C.); andrea.cinnirella@unimi.it (A.C.); federica.fornaro@unimi.it (F.F.); claudia.gallana@unimi.it (C.G.); piermario.perrone@unimi.it (P.M.P.); viktoriia.shishmintseva@unimi.it (V.S.); elena.pariani@unimi.it (E.P.); Silvana.Castaldi@unimi.it (S.C.); 2 Quality Unit Fondazione IRCCS Ca’ Granda OMP, 20122 Milan, Italy; Ester.Luconi@studenti.unimi.it

**Keywords:** influenza, health personnel, vaccination, vaccination coverage, vaccination refusal

## Abstract

*Background:* Despite recommendations, the influenza vaccination coverage rate in healthcare workers (HCWs) in Italy is far from the recommended target. The aim of the study is to analyze the influenza vaccination campaign performed in 2019 in a research and teaching hospital in Milan. *Methods:* The vaccination strategy included an ad hoc ambulatory, as in the previous years, and an onsite ambulatory, introduced for the first time. Personal data and professional categories were collected and analyzed using univariate logistic regression. HCWs who refused the vaccination were asked to fill in a questionnaire to explain their reasons for dissent. *Results:* The achieved vaccination coverage rate (VCR) for HCWs was 21.5 %, compared to 17.1% in 2018. The lowest VCR was registered among nurses (11.9%), while physicians had the highest VCR (40.7%). Prevalence ratios show that some professional categories were more frequently vaccinated for the first time than attending physicians (reference category); those with statistically significant confidence intervals were nurses (PR: 2.42; 95% CI: 1.78–3.28), residents (PR: 1.85; 95% CI: 1.36–2.53), and auxiliary staff (PR: 2.33; 95% CI: 1.45–3.74). *Conclusions:* An onsite vaccination strategy failed in providing a remarkable increase in VCR in 2019, but it is important to point out that the campaign was influenced by several logistic problems.

## 1. Introduction

Seasonal flu represents a major public health issue and an important cause of morbidity and mortality [1]. The World Health Organization (WHO) estimates that, worldwide, the annual influenza epidemic results in 3–5 million cases of severe illness and 290,000 to 650,000 deaths [2]. Health care workers (HCWs) are exposed to an increased risk of contracting flu and spreading it to vulnerable patients and colleagues when compared to the general population [3,4]. Influenza vaccination of HCWs is the most effective public health strategy to prevent influenza transmission in hospitals and to reduce work absenteeism [5,6]. Despite the fact that most of the international public health associations suggest it as a major preventive strategy [7], the vaccination rate is still very low among HCWs. When considering the vaccination coverage rate (VCR) on a worldwide level, there is a vast heterogeneity. In the countries where it is mandatory, as in the United States (US), the VCR is consistently higher than in those where it is not, as in Europe. In Italy, the recommended VCR target among HCWs is 75% but the observed one amounts to only 15.6% [8,9]. According to Legislative Decree n. 81 (9 April 2008), the Italian Government recommends the active distribution of influenza vaccine to HCWs every year during the influenza season from October to December [10]. Several studies have already addressed the issue as there is great interest in understanding the reasons why HCWs refuse vaccination in order to increase vaccination coverage rates. Data show that the reasons vary widely in accordance with profession, personal belief, age, gender, social impact, and access to the vaccination [11,12,13,14,15]. The aim of this study is to evaluate the compliance of HCWs to influenza vaccination in a research and teaching hospital in Milan (Italy) to analyze the reasons for the lack of improvement in the VCR that was expected with the introduction of an onsite vaccination strategy, and to discuss possible methods to improve adherence to future influenza vaccination campaigns.

## 2. Materials and Methods 

The 2019 influenza vaccination campaign for HCWs in a research and teaching hospital in Milan, where the research was conducted from 13 November to 20 December. The information needed by HCWs to access vaccination was guaranteed through the intranet system of the hospital, as in the previous years. This source of information was regularly updated. The planned calendar consisted of two different vaccination strategies: ad hoc ambulatory and onsite ambulatory. The ad hoc ambulatory was placed in the same location through the campaign and was open every Tuesday and Wednesday from 10 a.m. until 3 p.m., as in past years. The onsite ambulatory was implemented for the first time in 2019 and consisted of a team of professionals visiting each building of the hospital, 13 in total, according to the availability of the staff. The areas of activity of the hospital include all medical specialties, while for surgery specialties, it has urology, otolaryngology, oculistic, neurology, vascular, thoracic, and maxillofacial departments, and a transplant unit for liver, lung, and kidney. The onsite ambulatory took place in each ward from 11 a.m. to 2 p.m. in order to cover two work shifts and hence facilitate access to the service. According to the predefined calendar, we planned to open the ad hoc ambulatory each Tuesday and Wednesday from 13 November to 18 December, 11 days in total. The onsite ambulatory was planned so as to visit each ward at least once and, if possible, two times. As the onsite ambulatory had to be organized according to the availability of the chief of the ward, it was difficult to plan a detailed calendar in advance, and it could only be scheduled week by week, with last-minute adjustments during the campaign. Information to HCWs about the possibility of getting vaccinated at the onsite ambulatory was delivered through the intranet system and by direct communication from the department chief. Due to contingencies, the initially planned calendar had to undergo modifications. The main problems we encountered during the vaccination campaign were related to the disorganization of vaccine supply and storage, including delayed provisioning and a broken refrigerator, which led to the loss of about 1000 doses and a temporary stop of the campaign. Because of this, the ad hoc ambulatory ended up being open only 9 days instead of the initially planned 11 days, and one of the 9 days was actually rescheduled on a different day from those initially planned. The schedule of the onsite ambulatory was also affected, leading to the cancellation of 10 onsite visits. Despite all these issues, onsite vaccination was delivered once in each and every building of the hospital, and twice in three buildings where both the number of HCWs and the demand were higher. However, it is important to point out that our schedule covered only two work shifts instead of three. The disorganization of vaccine provisioning also led to a major change in the schedule of our campaign: its beginning was postponed to 13 November, even though the national board recommends a start in the last week of October. Those who received vaccination were asked to fill in a form to collect personal data, professional category (physician, nurse, student, administrative, researcher, and others), ward, previous influenza vaccination versus not, chronic diseases, previous vaccination, and informed consent. During onsite visits, part of the team encouraged the possibility of receiving free vaccinations at the temporary onsite ambulatory with a “door to door” approach, actively recruiting HCWs in their wards. The goal was to involve as many people as possible, taking the time to explain to them the importance of vaccination as a prevention strategy and answering their doubts and questions. 

An anonymous and self-administered form was used to assess the reasons for refusal of vaccination and the age, gender, specialty, and professional category of those who refused it. According to the recommendation of the regional health authority, the administered vaccine was Vaxigrip Tetra^®^, an inactivated tetravalent split vaccine injected intramuscularly in the deltoid muscle. All collected data were registered in a regional database managed by ATS Milan. Age was recorded as a continuous variable and calculated with median and interquartile ranges; subsequently, for statistical analysis, it was categorized in classes, and for each class number of subjects, percentages were reported. Other variables were categorical; thus, the number of subjects and percentages were reported. Data were analyzed using univariate logistic regression with a log link, and model results are reported as prevalence ratios with a 95% confidence interval. The likelihood ratio was used to test the general association between the vaccination site and the demographic characteristics, occupation, and area of activity of the subjects. Different logistic models were used to analyze the association of the following: (1) site of vaccination for all vaccinated people, (2) site of vaccination for people who received their first vaccination in the 2019 campaign, and (3) the attitude of previous vaccination. The analyses were performed using software R, release 3.5.1 (R core team 2018). 

No ethical approval was required for this study, according to Regional Law No.3 of the year 2012 of the Lombardy Region.

## 3. Results

Table 1 shows data about vaccinated HCWs in 2018 and 2019 out of the total number of HCWs working at the hospital. Data were provided by the human resources office. In 2019, we vaccinated 733 HCWs, 238 residents, 166 students, and 16 volunteers for a total of 1153 subjects. The achieved vaccination coverage rate (VCR) for HCWs was 21.5% in 2019 (733/3405) as compared to 14.5% in 2018 (495/3417). Our campaign showed an increase of VCR among HCWs compared to the previous year (21.5% versus 14.5%). The lowest VCR was recorded among nurses (11.9%), followed by administrators (13.9%) and technicians (22.2%). Attending physicians reported the highest VCR (40.7%). The area of activity with the highest number of vaccinated HCWs was medical specialty (374/1153; 32.4%), and the majority of HCWs who were vaccinated were 19–39 years (642/1153; 55.7%). Although nurses still present a very low VCR compared to medical staff, it is interesting to observe that their VCR doubled when compared to the previous year (11.9% versus 6.2%), while the medical staff showed only a slight increase (40.7 versus 34.2%).

Table 2 shows detailed data about vaccinated HCWs in 2018 and 2019. For 2019, we collected more data as compared to 2018. The population considered is the total number of vaccinated people. 

Table 3 shows the results of the logistic regression model: the prevalence ratio compares onsite versus ad hoc ambulatory vaccination with a 95% confidence interval and likelihood ratio test. The professional categories that were more frequently vaccinated at the onsite ambulatory compared to the physicians (reference category) were residents (PR: 1.35; 95% CI: 1.13–1.60), auxiliary staff (PR: 1.64; 95% CI: 1.27–2.11) and volunteers (PR: 1.59; 95% CI: 1.12–2.28), while for the area of activity, the analysis shows a higher prevalence ratio for the pediatric area (PR: 1.70; 95% CI: 1.38–2.08) and the ICU area (PR: 1.40; 95% CI: 1.07–1.81) than the general medicine area (reference category). 

The likelihood ratio test shows a significant association between the vaccination site and all the other variables except for sex.

Table 4 shows detailed data about people who have vaccinated before versus never vaccinated before. An interesting piece of data to point out is that the largest percentage of HCWs who received the influenza vaccination for the first time was registered at the onsite ambulatory as opposed to the ad hoc ambulatory: 226/514 (44%) versus 150/639 (23.5%). 

Table 5 shows the results of the logistic regression model, namely, the prevalence ratios comparing never vaccinated before versus vaccinated before, with 95% confidence intervals and likelihood ratio tests.

Some professional categories were more frequently vaccinated for the first time compared to physicians (reference category). More specifically, those with statistically significant confidence intervals were nurses (PR: 2.42; 95% CI: 1.78–3.28), residents (PR: 1.85; 95 %CI 1.36–2.53), auxiliary staff (PR: 2.33; 95% CI 1.45–3.74) and other (PR: 1.53; 95% CI 1.02–2.30).

The total number of refusals was 374; data on their professional category and reasons for refusal are shown in Table 6. Since it was possible to state more than one reason, the total number of answers overcomes the total number of questionnaires. Out of 374 subjects who refused vaccination, 285 were females (76.2%) and 84 males (22.5%); the fact that nurses are mostly females contributes to the gender discrepancy [16,17]. The most common motivations identified by our study are a young age, the belief that flu is not a serious disease, the concern about side effects, and the belief that vaccination is not an effective means of prevention. It is interesting to note that 49/375 (13.1%) of HCWs reported fear of side effects as the reason for refusal, even though influenza vaccination has been long recognized as a safe means of prevention by the main scientific associations. Among the 22 HCWs who reported a medical reason as the motivation for refusal, some referred their true motivations to be autoimmune disease or assumption of contraindicated drugs, while others referred to unreasonable motivations, such as a hepatitis C virus (HCV) infection. 

## 4. Discussion

Although we implemented our vaccination campaign with an onsite vaccination strategy, it failed to provide a remarkable increase in VCR, as shown by a similar experience in another Italian hospital [18]. The VCR increased by 6%; however, such an increase was not homogeneous. Although physicians are still the professional category with the highest VCR, introducing an onsite vaccination strategy was more effective on nurses, residents, and auxiliary staff, especially when considering first-time vaccination. Our results are deeply influenced by the logistics problem that we encountered, and by the lack of a proper promotional campaign to inform HCWs about the possibility of getting vaccinated at the onsite ambulatory. However, our results support the evidence of the efficacy of the onsite strategy in reaching those HCWs who usually do not actively search for this service and highlight the need to implement different strategies to involve different populations. Our results regarding the reasons for vaccine hesitancy are in line with several other studies [13,19,20,21], but there are several other motivations why HCWs refuse vaccination other than those aforementioned. For instance, a study [22] showed that some people believe that the mutation of the virus and mismatch of vaccine strains could be reasons for vaccine ineffectiveness, while others suggest that current scientific evidence is not sufficient to support vaccination programs. Many HCWs expressed concern about possible side-effects of the vaccine, including both mild influenza-like symptoms and discomfort at the injection site, and more serious symptoms such as Guillain–Barré syndrome. This study also highlights wrong beliefs, such as the conviction that natural remedies are more effective means of prevention [22]. When collecting the refusal forms, we also had the opportunity to discuss these beliefs regarding influenza vaccination with each HCW, providing us with information about several other factors that should be taken into account in order to understand the reasons for the failure of our campaign. The major reason for complaint concerned the disorganization of the ad hoc ambulatory and the long waiting queue deriving from it. Additionally, they complained about last-minute closures and about the lack of information on the plans of the onsite ambulatory. 

Some of them even suggested that the vaccination was provided in order to bring economic benefits to pharmaceutical companies, while others surprised us with very peculiar answers such as “I use antiviral homeopathic vaccines for flu and I never get sick” or “the vaccine is useful only for private workers”. 

Those who were skeptical about vaccination often told us that they were not at risk of getting the flu or did not consider vaccination safe nor effective. When analyzing the data, we should keep in mind that approximately 80 workers refused to fill in the dissent forms, according to our estimate. 

## 5. Conclusions

In conclusion, our vaccination campaign failed on several levels. We analyzed all the issues that came up, focusing on possible improvements for future campaigns. For the influenza vaccination campaign of 2020, we would like to implement several strategies, learning from the problems that we encountered this year. First of all, during the first phase of the campaign, we should implement a number of ad hoc ambulatories and increase their opening hours in order to cope with the higher requests that coincide with the first two weeks. Furthermore, it would be important to have an onsite ambulatory that covers all the shift work, meaning that it should be offered at least 3 times per day in each ward, possibly avoiding high-peak working hours. Nevertheless, such ambulatories should be planned and sponsored more efficiently.

To increase the willingness of HCWs to get vaccinated, we could trigger competition between the different wards, a strategy that has already been tested and proven effective [23,24]. Furthermore, we would like to develop educational videos that explain the benefits of vaccination and fight the most common false beliefs about it and send them to all hospital workers via the intranet. Videos should be clear and attractive, using infographics and images, with a key role model from the staff showing the right example. It is important to remember that several factors should be taken into account when assessing the reasons why HCWs accept or refuse influenza vaccinations. As showed by To K.W. et al., the choice to vaccinate or not is influenced by a complex interplay between individual, organizational, and social factors [19]. The difficulties encountered, as well as the measures required to ensure the achievement of the recommended goals for VCR, tend to vary, as all the abovementioned factors bear a different weight, depending on the circumstances. In order to develop an effective strategy, it is of paramount importance to tailor the intervention to address the peculiarities of our own context. 

## Figures and Tables

**Table 1:** Comparison between vaccination coverage rate for healthcare workers, 2018 versus 2019.

Health Care Workers	Vaccinated in 2019/Total Number of Employers	Vaccinated in 2018/Total Number of Employers
Medical staff	283/696 (40.7%)	237/692 (34.2%)
Nurse staff	171/1431 (11.9%)	89/1436 (6.2%)
Administrative staff	48/345 (13.9%)	42/351 (12%)
Technician staff	96/433 (22.2%)	71/435 (16.3%)
Other staff	135/500 (27%)	56/503 (11.1%)
Total	733/3405 (21.5%)	495/3417 (14.5%)

**Table 2:** Data about vaccinated people out of the total vaccinated (year 2019).

Total Population Vaccinated	2019			2018
	Ad Hoc Ambulatory = 639	On Site Vaccination = 514	Total = 1153	Ad Hoc Ambulatory = 759
**Gender N (%)**				Data not available
Male	214 (33.5%)	199 (38.7%)	413 (35.8%)	
Female	425 (66.5%)	315 (61.3%)	740 (64.2%)	
**Age**				Data not available
Median, IQR	40, 28	32, 20	36, 25	
19–39 (N) (%)	(315) (49.3%)	(327) (63.6%)	(642) (55.7%)	
40–59 (N) (%)	(236) (36.9%)	(138) (26.8%)	(374) (32.4%)	
60–80 (N) (%)	(88) (13.8%)	(49) (9.5%)	(137) (11.9%)	
**Occupation**				
Physician	161 (25.2%)	122 (23.7%)	283 (24.5%)	237 (31.2%)
Resident	100 (15.6%)	138 (26.8%)	238 (20.6%)	136 (17.9%)
Student	102 (16.0%)	64 (12.5%)	166 (14.4%)	103 (13.57%)
Nurse	82 (12.8%)	89 (17.3%)	171 (14.8%)	89 (11.7%)
Other	62 (9.7%)	38 (7.4%)	100 (8.7%)	56 (7.4%)
Technician	80 (12.5%)	16 (3.1%)	96 (8.3%)	71 (9.4%)
Administrative	36 (5.6%)	12 (2.3%)	48 (4.2%)	42 (5.5%)
Auxiliary staff	10 (1.6%)	24 (4.7%)	34 (2.9%)	Data not available
Volunteer	5 (0.8%)	11 (2.1%)	16 (1.4%)	25 (3.3%)
“NA”	1 (0.2%)	0 (0.0%)	1 (0.1%)	
**Area of Activity**				Data not available
Administrative	21 (3.3%)	6 (1.2%)	27 (2.3%)	
Newborn Area	51 (8%)	46 (8.9%)	97 (8.4%)	
Pediatric Area	38 (5.9%)	79 (15.4%)	117 (10.1%)	
General Surgery	24 (3.8%)	20 (3.9%)	44 (3.8%)	
Specs Surgery	90 (14.1%)	80 (15.6%)	170 (14.7%)	
General Medicine	136 (21.3%)	90 (17.5%)	226 (19.6%)	
Specs Medicine	232 (36.3%)	142 (27.6%)	374 (32.4%)	
Intensive Care Unit	32 (5%)	40 (7.8%)	72 (6.2%)	
Other	10 (1.6%)	5 (1%)	15 (1.3%)	
NA	5 (0.8%)	6 (1.2%)	11 (1.0%)	

**Table 3:** Prevalence ratios of onsite ambulatory versus ad hoc ambulatory (year 2019).

Variable	PR (95% C.I)	X^2^ Test (Likelihood)
**Gender**		
Female	Reference	0.06604
Male	1.13 (0.99–1.29)	
**Age**		
19–39	Reference	<0.000001
40–59	0.72 (0.62–0.84)	
60–80	0.70 (0.55–0.89)	
**Profession**		
Physician	Reference	<0.000001
Resident	1.35 (1.13–1.60)	
Student	0.89 (0.71–1.13)	
Nurse	1.21 (0.99–1.47)	
Other	0.88 (0.66–1.17)	
Technician	0.39 (0.24–0.62)	
Administrative	0.58 (0.35–0.96)	
Auxiliary staff	1.64 (1.27–2.11)	
Volunteer	1.59 (1.12–2.28)	
**Area of activity**		
General Medicine	Reference	<0.000001
Administrative	0.56 (0.27–1.15)	
Newborn Area	1.19 (0.91–1.55)	
Pediatric Area	1.70 (1.38–2.08)	
General Surgery	1.14 (0.80–1.64)	
Specs Surgery	1.18 (0.94–1.48)	
Specs Medicine	0.95 (0.78–1.17)	
Intensive Care Unit	1.40 (1.07–1.81)	
Other	0.84 (0.40–1.74)	

**Table 4:** Never vaccinated before versus vaccinated before (year 2019).

	Never Vaccinated before (N = 376)	Vaccinated before (N = 777)	Total (N = 1153)
**Gender N (%)**			
Male	118 (31.4%)	295 (38.0%)	413 (35.8%)
Female	258 (68.6%)	482 (62.0%)	740 (64.2%)
**Age**			
Median, IQR	30.00, 20	40.00, 26	36, 25
19–39 (N) (%)	(261) (69.4%)	(381) (49.0%)	(642) (55.7%)
40-59 (N) (%)	(95) (25.3%)	(279) (35.9%)	(374) (32.4%)
60-80 (N) (%)	(20) (5.3%)	(117) (15.1%)	(137) (11.9%)
**Profession**			
Physician	50 (13.3%)	233 (30.0%)	283 (24.5%)
Resident	78 (20.7%)	160 (20.6%)	238 (20.6%)
Student	93 (24.7%)	73 (9.4%)	166 (14.4%)
Nurse	73 (19.4%)	98 (12.6%)	171 (14.8%)
Other	27 (7.2%)	73 (9.4%)	100 (8.7%)
Technician	25 (6.6%)	71 (9.1%)	96 (8.3%)
Administrative	13 (3.5%)	35 (4.5%)	48 (4.2%)
Auxiliary staff	14 (3.7%)	20 (2.6%)	34 (2.9%)
Volunteer	3 (0.8%)	13 (1.7%)	16 (1.4%)
“NA”	0 (0.0%)	1 (0.1%)	1 (0.1%)
**Area of Activity**			
Administrative (10)	7 (1.9%)	20 (2.6%)	27 (2.3%)
Newborn Area (11)	32 (8.5%)	65 (8.4%)	97 (8.4%)
Pediatric Area (12)	41 (10.9%)	76 (9.8%)	117 (10.1%)
General Surgery (13)	14 (3.7%)	30 (3.9%)	44 (3.8%)
Specs Surgery (14)	41 (10.9%)	129 (16.6%)	170 (14.7%)
General Medicine (15)	93 (24.7%)	133 (17.1%)	226 (19.6%)
Specs Medicine (16)	109 (29.0%)	265 (34.1%)	374 (32.4%)
Intensive Care Unit (17)	30 (8.0%)	42 (5.4%)	72 (6.2%)
Other (19)	5 (1.3%)	10 (1.3%)	15 (1.3%)
NA	4 (1.1%)	7 (0.9%)	11 (1.0%)

**Table 5:** Prevalence ratios of never vaccinated before versus vaccinated before (year 2019).

Variable	PR (95% C.I)	X^2^ Test (Likelihood)
**Gender**		
Female	Reference	0.02798
Male	0.82 (0.68–0.98)	
**Age**		
19–39	Reference	<0.000001
40–59	0.62 (0.51–0.76)	
60–80	0.36 (0.24–0.54)	
**Profession**		
Physician	Reference	<0.000001
Resident	1.85 (1.36–2.53)	
Student	3.17 (2.38–4.22)	
Nurse	2.42 (1.78–3.28)	
Other	1.53 (1.02–2.30)	
Technician	1.47 (0.97–2.24)	
Administrative	1.53 (0.90–2.60)	
Auxiliary staff	2.33 (1.45–3.74)	
Volunteer	1.06 (0.37–3.03)	
**Area of activity**		
General Medicine	Reference	
Administrative	0.63 (0.33–1.21)	0.0167
Newborn Area	0.80 (0.58–1.11)	
Pediatric Area	0.85 (0.64–1.14)	
General Surgery	0.77 (0.49–1.22)	
Specs Surgery	0.59 (0.43–0.80)	
Specs Medicine	0.71 (0.57–0.88)	
Intensive Care Unit	1.01 (0.74–1.39)	
Other	0.81 (0.39–1.69)	

**Table 6:** Reasons for refusal (year 2019).

Rejection Data	Total = 374
**Gender N (%)**	
Male	84 (22.5%)
Female	285 (76.2%)
NA	5 (1.3%)
**Age**	
Median (IQR)	47 (19.25)
19–39 N (%)	127 (34.0%)
40-59 N (%)	214 (57.2%)
60-80 N (%)	27 (7.2%)
NA N (%)	6 (1.6%)
**Area of Activity**	
Administrative	10 (2.7%)
Newborn	31 (8.3%)
Pediatric	39 (10.4%)
General Surgery	40 (10.7%)
Specs Surgery	51 (13.6%)
General Medicine	59 (15.8%)
Specs Medicine	103 (27.5%)
Intensive Care Unit	29 (7.8%)
NA	12 (3.2%)
**Profession**	
Physician	52 (13.9%)
Resident	12 (3.2%)
Student	19 (5.1%)
Nurse	149 (39.8%)
Other	20 (5.3%)
Technician	50 (13.4%)
Administrative	21 (5.6%)
Auxiliary staff	40 (10.7%)
Volunteer	2 (0.5%)
NA	9 (2.4%)
**Reasons for refusal**	
I’m young and/or I don’t think I will be sick	83
Flu isn’t a severe illness therefore I don’t think mandatory vaccination is necessary	65
I recommend the vaccination to my patients but I will refuse it for myself	54
I’m afraid of side effects	49
I’ve vaccinated in previous years but I have caught influenza nevertheless	38
I don’t think vaccination is an effective preventive technique	35
Side effects during previous influenza vaccination	29
Medical reasons	22
I have no time	13
Other	9
I would like get vaccinated only one time and not each year	8
I was vaccinated for influenza in the last few years	7
